# In-hospital mortality of patients requiring unplanned postoperative ventilatory support: a multicenter observational study

**DOI:** 10.1186/s13741-022-00276-x

**Published:** 2022-08-23

**Authors:** Ron Magor, Inbal Dabush-Elisha, Daniel Aviram, Dana Karol, Adi Syn-Hershko, Reut Schvartz, Barak Cohen, Idit Matot

**Affiliations:** 1 Division of Anesthesia, Intensive Care, and Pain Management, Tel-Aviv Medical Center, Tel-Aviv University, 6423906 Tel-Aviv, Israel; 2 Department of Anesthesia, Edith Wolfson Medical Center, Tel-Aviv University, Holon, Israel; 3 Sanz Medical Center — Laniado Hospital, Tel-Aviv University, Netanya, Israel; 4 Outcomes Research Consortium, Cleveland Clinic, Cleveland, OH USA

**Keywords:** Postanesthesia care unit, Mechanical ventilation, Complications, Postoperative, Complications, Postoperative pulmonary

## Abstract

**Background:**

Most patients who are admitted non-intubated to surgery are extubated at surgery conclusion. Yet, 1–2% require unplanned postoperative ventilatory support. The outcome of these patients has not been thoroughly evaluated to date and is the focus of the present study.

**Methods:**

Two-center observational study assessing characteristics and outcomes of surgical patients with unplanned mechanical ventilation during PACU stay between 2017 and 2019. Patients who arrived intubated to the operating room or were transferred directly to the intensive care unit (ICU) were excluded. The co-primary aims were to assess overall in-hospital mortality and to compare mortality between patients who were extubated in PACU and those who were discharged from PACU still intubated. The secondary aims were to compare postoperative respiratory infection and unplanned admissions to the ICU. Multivariate logistic regression was used to compare the groups and adjust for potential confounding variables.

**Results:**

Overall, 698 patients were included. Of these, 135 died during hospital stay (mortality rate 19.3%, compared with 1.0% overall postoperative in-hospital mortality). Patients who still required ventilatory support at PACU discharge were significantly sicker, majority needed emergency surgery, and had more complicated surgical course compared to those who were extubated in PACU. In addition, their mortality rate [36% vs. 9%, adjusted *OR* (95% *CI*) 5.8 (3.8–8.8), *p* < 0.001], postoperative respiratory infection, and unplanned admission to ICU rates were also significantly higher.

**Conclusion:**

Unplanned postoperative mechanical ventilation is associated with noteworthy morbidity and mortality, with significantly higher rates in those of need for protracted (vs. short) mechanical ventilation. The remarkable mortality rate in patients extubated shortly after arriving to the PACU emphasizes the need for further studies to explore prompting factors and whether we can intervene to improve patients’ outcome.

## Introduction

More than 300 million surgical procedures are performed annually worldwide [Weiser et al. 2016]. The majority are performed under general anesthesia, frequently involving endotracheal intubation. While most patients are extubated at surgery conclusion, some remain intubated unexpectedly [Schultz et al. 2017] and are transferred to the postanesthesia care unit (PACU) still intubated. Patients who require unplanned ventilatory or airway support after surgery are in most cases different than patients who are suitable for extubation, either on baseline morbidities or in terms of surgical and anesthetic complexity [Schultz et al. 2017]. Some need relatively short-term support, as in the case of excessive intraoperative administration of opioids or neuromuscular blocking agents, and are extubated while in the PACU. Others, such as those with massive intraoperative bleeding, severe metabolic abnormalities, or cardiac/neurologic injury, need longer ventilatory support and are usually transferred to the intensive care unit (ICU). Unplanned postoperative mechanical ventilation is generally considered a severe complication, but the long-term outcome of patients with unplanned need for short or long period of postoperative ventilatory support is largely unknown.

We therefore aimed to report the outcomes of patients who required unplanned mechanical ventilation after surgery. We also aimed to compare the incidence of postoperative respiratory infections, unplanned ICU admission, and death between those who underwent extubation in PACU and those who remained intubated after PACU discharge.

## Methods

We conducted a 2-center observational study of all surgical patients with unplanned mechanical ventilation during PACU stay. After receiving institutional review board approvals from both sites [Tel-Aviv Sourasky Medical Center in Tel-Aviv (TLVMC) and Edith Wolfson Medical Center in Holon (WMC)], data were collected from the medical records of eligible patients. The need for individual consent was waived by the institutional review board of both sites due to the retrospective nature of the analysis.

We included all patients admitted to the PACU that were mechanically ventilated at any time during their PACU stay between June 2017 and June 2019 and December 2017 to November 2018 for TLVMC and WMC, respectively. We excluded patients who were scheduled to remain intubated postoperatively, those that were transferred from the operating room (OR) directly to the ICU, and patients who were intubated before arriving to the OR. Patients who were admitted to PACU without having surgery (such as admission for impending airway observation) were also excluded.

All anesthesia, surgery, PACU, and postsurgical hospitalization data were reviewed. The study had two pre-defined primary aims: first, to describe the overall mortality of patients who require mechanical ventilation during their PACU stay, and, second, to compare mortality risk between patients who were extubated in PACU and those who were discharged from PACU while still intubated. Secondary aims were to compare the risk of unplanned ICU admission and postoperative respiratory infections between patients who were extubated in PACU and those who were not. We also evaluated a composite outcome of all 3 outcomes (unplanned admission to ICU, respiratory infection, and mortality).

The clinical practice in our hospitals is that patients planned for postoperative ICU admission are transferred directly from the OR to the ICU without being admitted to PACU. Hence, all patients who were mechanically ventilated in PACU (either remained intubated after surgery or re-intubated in PACU), and then transferred to an ICU, were considered unplanned ICU admissions. Postoperative respiratory infection was defined as any new postoperative diagnosis of pneumonia or pneumonitis.

### Statistical analysis

Dichotomous variables are presented as *n* (%) and were analyzed with chi-square or Fisher’s exact tests. Continuous variables are presented as mean (standard deviation, SD) and analyzed by 2-tailed *t*-test if normality is assumed or as median [interquartile range (IQR)] and analyzed with the Mann-Whitney test if non-normally distributed. Multivariate logistic regression was used to assess the primary hypothesis of association between extubation status in PACU as the independent variable and mortality as the dependent variable. All variables with *P* < 0.1 or those with clinical importance as potential confounders were entered into the model. Similar models were built for the secondary analyses, which were adjusted for multiple outcomes by the Bonferroni correction and thus reported as hazard ratios with 97.5% confidence intervals. The exploratory composite outcome was analyzed in a similar fashion.

We used a convenience sample of all eligible patients during the study period, so no formal sample size calculation was conducted. A post hoc power justification found that with a mortality rate of 9% in patients extubated in PACU, and a sample of 270 patients not extubated, we could find an increase in mortality risk of about 5% with a significance level of 0.05 and power of 80%. All analyses were performed with SPSS version 25.0.

## Results

### Patients

In total, 701 patients had unplanned mechanical ventilation in PACU during the study period and were therefore included in the analysis. Of these, 477 were from TLVMC, and 224 were from WMC. These patients represent about 1–2% of all noncardiac surgical cases during the study period (48,000 and 13,000 noncardiac surgeries in TLVMC and WMC during the relevant study periods, respectively). Details regarding extubation in PACU were not available for 3 patients, and they were excluded from further analyses. Forty patients (5.7%) were admitted to PACU not intubated and were intubated during PACU stay, after an average of 137 min.

Average (SD) age was 67 (18) years, and 49% was male. Fifty-seven percent of the patients (*n* = 401) had undergone urgent or emergent surgery, a third were classified as American Society of Anesthesiologists’ (ASA) physical scores 4 or 5, and more than half had abdominal surgery. Two-hundred and seventy-five cases (39%) started after regular working hours, and 418 (60%) cases ended during on-call hours. Further demographic, anesthetic, surgical, and postoperative data are provided in Table 1.

**Table 1:** Patient characteristics

	Extubated (*N* = 429)	Not extubated (*N* = 269)	*p*-value
Age, years	66 (17)	68 (17)	0.226
BMI, kg·m^−2^	27.3 (6.7)	27.3 (6.8)	0.937
Female sex	236 (55%)	117 (43%)	**0.003**
ASA physical status			**< 0.001**
I	17 (4%)	12 (4%)	
II	142 (33%)	34 (13%)	
III	180 (42%)	90 (33%)	
IV	87 (20%)	118 (44%)	
V	2 (0.5%)	15 (6%)	
Active smoking	99 (23%)	68 (25%)	0.518
Active infection	47 (11%)	78 (29%)	**< 0.001**
Urgent/emergent surgery	200 (47%)	201 (75%)	**< 0.001**
Weekend and/or holiday	48 (11%)	55 (20%)	**0.001**
Evening and/or night hours	242 (56%)	173 (64%)	**0.042**
Surgical department			**< 0.001**
General surgery	169(39%)	112 (42%)	
Orthopedic surgery	59 (14%)	25 (9%)	
NORA	46 (11%)	26 (10%)	
Gynecologic surgery	40 (9%)	2 (1%)	
ENT	34 (8%)	16 (6%)	
Neurosurgery	23 (5%)	32 (12%)	
Urology	22 (5%)	13 (5%)	
Vascular surgery	12 (3%)	20 (7%)	
Thoracic surgery	11 (3%)	12 (4%)	
Other^a^	8 (2%)	8 (3%)	
Surgical approach — laparotomy/thoracotomy, *n* (%)	129 (30%)	106 (39%)	**0.011**
Anesthesia duration, minutes	246 [133, 330]	206.3 [116, 251]	**< 0.001**
Hemodynamic compromise^b^	234 (54%)	159 (59%)	0.237
Respiratory compromise^c^	61 (14%)	66 (24%)	**0.001**
Intraoperative blood products administration	77 (18%)	71 (26%)	**0.016**
Intraoperative vasopressor administration	20 (5%)	36 (13%)	**< 0.001**
Hypothermia on PACU admission^d^	60 (14%)	35 (13%)	0.715
Intraoperative crystalloids, L	2.1 [1.1, 3.1]	2.2 [1.1, 3.6]	**0.032**

Demographic, medical, anesthetic, and surgical characteristics of patients who were mechanically ventilated in PACU (*N* = 698) and were either extubated in PACU or discharged from PACU still intubated (not extubated). Data presented as mean (standard deviation), *n* (%), or median [interquartile range], as appropriate. All variables that were significantly different between the groups are marked in bold and were entered into the statistical model and adjusted for in the multivariate analysis.

^a^all departments with frequency < 3%, combined

^b^hemodynamic compromise defined as either systolic blood pressure < 90 mmHg or mean arterial pressure < 65 mmHg that initiated a pharmacologic intervention or transfusion

^c^respiratory compromise defined as an intraoperative respiratory event that required pharmacologic intervention, recruitment maneuver, or increase in the fraction of inspired oxygen to 0.8 or greater for at least 2 min

^d^hypothermia defined as core temperature < 35° centigrade

*ASA* American Society of Anesthesiologists, *BMI* body mass index, *ENT* ears, nose, and throat, *NORA* nonoperating room anesthesia, *PACU* postanesthesia care unit

### Primary outcomes

Nearly one-fifth of all patients who were mechanically ventilated in PACU died during hospitalization (in-hospital mortality *n* = 135, 19.3%), of whom 4 patients (0.6%) died in PACU.

**Fig. 1:**
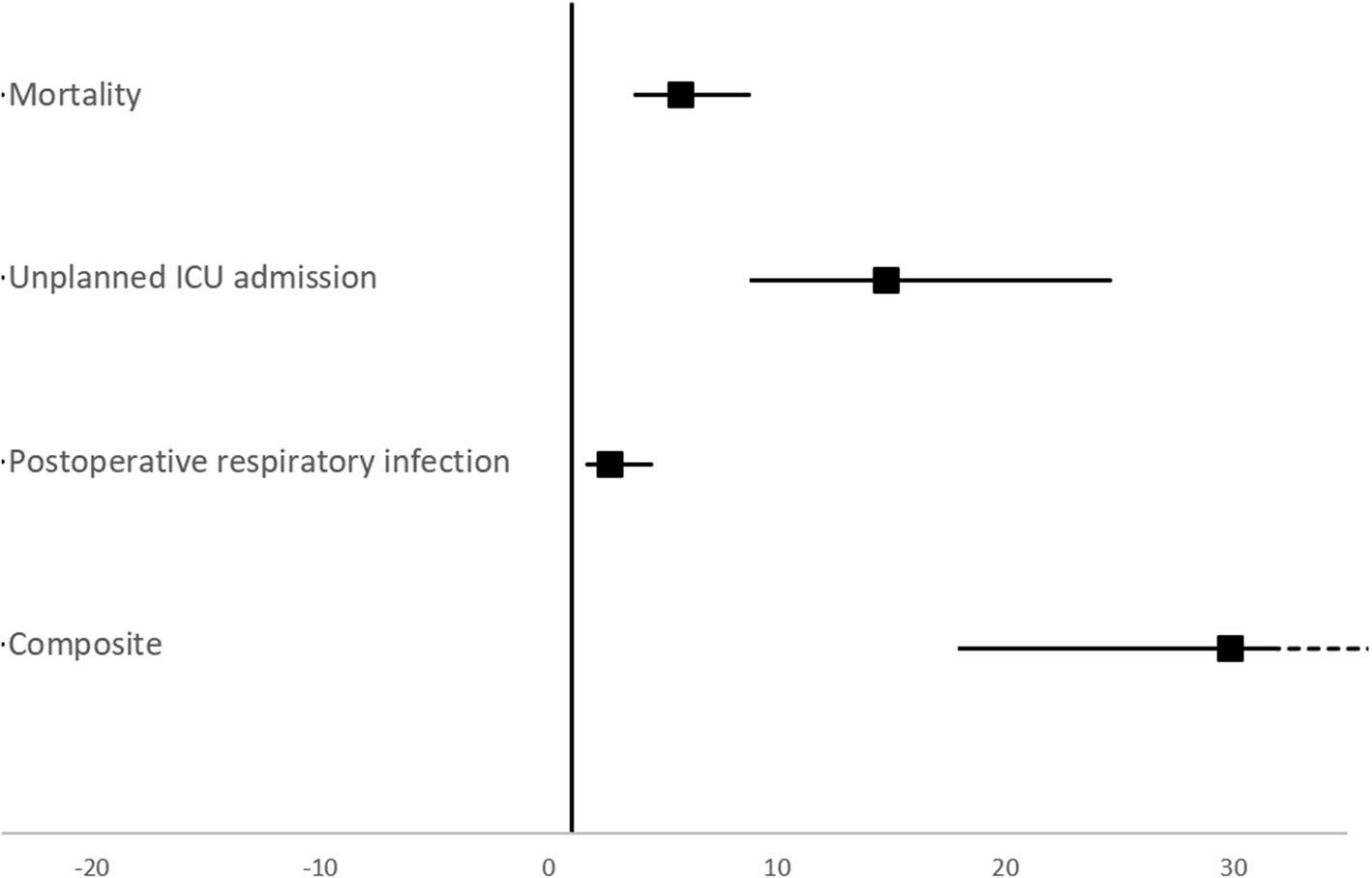
Forest plot of the odds ratio (95% confidence intervals) of the primary and secondary outcomes comparing patients who were still intubated at PACU discharge and those who were extubated during PACU stay. ICU, intensive care unit; PACU, postanesthesia care unit

Of the 698 study patients, 269 patients (39%) were not extubated in PACU. Compared with the 429 patients (61%) who were extubated in PACU, they had a significantly higher ASA score, increased odds of having urgent/emergent surgery and evening/weekend surgery, and increased risk of intraoperative vasopressor administration and respiratory compromise (Table 1). After adjustment for potential confounders, patients who remained intubated after PACU discharge had a significantly higher mortality rate (36% compared with 9%, adjusted *OR* (95% *CI*) 5.8 (3.8–8.8), *p* < 0.001, Table 2, Fig. 1).

**Table 2:** Main clinical outcomes

Outcome	All patients (*N* = 698)	Extubated in PACU (*N* = 429)	Not extubated in PACU (*N* = 269)	
Primary outcome				**aOR (95%** ***CI*)**
In-hospital mortality	135 (19%)	38 (9%)	97 (36%)	5.8 (3.8–8.8)
Secondary outcomes				**aOR (97.5%** ***CI*)**
Unplanned ICU admission	317 (45%)	97 (23%)	220 (82%)	14.8 (8.9–24.6)
Postoperative respiratory infection	97 (14%)	39 (9%)	58 (22%)	2.7 (1.7–4.5)
Composite outcome				**aOR (95%** ***CI*)**
Mortality, ICU admission, or respiratory infection		131 (30.5%)	250 (92.9%)	29.9 (18.0–49.8)

Comparison of the primary, secondary, and exploratory outcomes between patients extubated during PACU stay versus remaining intubated beyond PACU discharge. Results reported as adjusted odds ratios and 95% (for the primary and exploratory outcomes) or 97.5% (Bonferroni, for the 2 secondary outcomes) confidence intervals.

*CI* confidence interval, *aOR* adjusted odds ratio, *ICU* intensive care unit, *PACU* postanesthesia care unit

### Secondary outcomes

Postoperative respiratory infections were significantly more common in patients who remained intubated vs. those extubated during PACU stay, with adjusted OR of 2.7 [97.5 *CI* 1.7, 4.5]. Unplanned admission to ICU was also significantly more common among patients not extubated in PACU (82% vs. 23%, adjusted *OR* 14.8 [8.9, 24.6]). The composite of the 3 outcomes (mortality, ICU admission, and respiratory infection) was similarly higher among patients not extubated in PACU (adjusted *OR* 29.9 [18.0, 49.8], Table 2, Fig. 1).

### Patients intubated in PACU

Of the subgroup of 40 patients who were re-intubated in PACU, 12 (30%) was later extubated (after 235 min on average) before being discharged from PACU. The rest (*n* = 28, 70%) was discharged to a high-dependency unit still intubated, for an average of 5.7 days of mechanical ventilation. The most common etiology for PACU re-intubation was respiratory failure (75%), and in-hospital mortality in this subgroup was 22.5% (*n* = 9).

## Discussion

To the best of our knowledge, this is the first thorough description of outcomes of such a large cohort of consequent patients who had unplanned mechanical ventilation during their PACU stay. This study provides data from 2 centers for a population of 698 patients undergoing surgery and requiring unplanned postoperative ventilatory support. Nearly 20% of included patients died before hospital discharge; mortality rate of 36% was found in those who needed protracted mechanical ventilation (beyond PACU stay) and 9% of patient with short need for mechanical support.

### Postoperative mechanical ventilation

It appears that in most reports, roughly 1–2% of surgical patients unexpectedly require postoperative ventilatory support. The LAS-VEGAS observational study reported an overall incidence of unexpected prolonged need of postoperative mechanical ventilation of about 1%, reaching 2.3% among patients at increased risk of postoperative pulmonary complications [Schultz et al. 2017]. Another observational study of 1200 patients undergoing major surgery also reported that 1.7% required prolonged postoperative mechanical ventilation/re-intubation [Fernandez-Bustamante et al. 2017]. This estimation is in agreement with our finding of need for mechanical ventilation at surgery conclusion in 1–2% of the total surgical volume during the relevant study period (*N* approximately 61,000, data not shown). Our current study adds to the available knowledge by reporting the outcomes of these specific 1–2% of surgical patients.

### Postoperative in-hospital mortality

The overall in-hospital mortality of adult patients recovering from surgery is highly variable, depending on the populations evaluated. The International Surgical Outcomes Study (ISOS) group reported postoperative in-hospital mortality of 0.5% when considering only elective inpatient noncardiac surgical cases [Pearse et al. 2016]. The European Surgical Outcomes Study (EuSOS) group reported a much higher mortality rate of 4% when urgent and emergency cases were also included in the analysis [Pearse et al. 2012]. In another report, mortality among patients undergoing major non-cardiac and cardiovascular surgery was as high as 8% [Ghaferi et al. 2009]. A mortality risk stratification model found that patients with severe comorbidities, having urgent high-risk surgery, can have a mortality rate exceeding 10% [Glance et al. 2012]. The present study reports a mortality rate twice as high for the overall population of patients admitted to the PACU with unplanned need for ventilatory support and more than three times higher for patients in need of mechanical ventilation beyond PACU stay. As anticipated, substantial differences in crude- and risk-adjusted mortality rates were identified between patients extubated during their PACU stay and those not extubated. The latter were significantly sicker, most of them had undergone urgent or emergent surgery, often during nighttime. Yet, the alarming 9% in-hospital mortality rate among patients who were extubated shortly after admission to PACU (compared with mortality rate of 1% among our patients who did not require postoperative ventilatory support) needs further investigations to explore preventable interventions.

### Postoperative complications

Postoperative complications are frequent and often severe [Bartels et al. 2013]. The incidence of complications after noncardiac surgeries is as high as 35% [Belcher et al. 2017]. Pulmonary complications are the most common. Even mild pulmonary complications are associated with longer duration of hospital and ICU stay, greater risk of ICU admission, and death [Fernandez-Bustamante et al. 2017]. The ARISCAT observational study reported an overall postoperative pulmonary complication rate of about 10% [Canet et al. 2010]. Specifically, respiratory infections occurred in 1.6% of patients. The LAS-VEGAS observational study [Schultz et al. 2017] also reported an overall 10% incidence of respiratory complications. Nonetheless, patients at increased preoperative risk for postoperative respiratory complications had nearly double that incidence (19%). Pneumonia was reported in 0.4% of patients, but incidence increased to 1.1% among high-risk patients. In the present study, significantly much higher incidence of respiratory infections (22%) among patients who remained intubated beyond PACU stay reflect the preoperative status of the patients (for example, almost 30% were admitted with active infection), the complicated perioperative course, and the need for prolonged intubation. Yet, 14% respiratory infection among patients extubated already in the PACU needs further investigation to comprehend potential causes and interventions that might assist in reducing the need for short postoperative ventilatory support. Similarly, whereas 8% of surgical inpatients recovering from noncardiac surgery across Europe [Pearse et al. 2012] were admitted to the ICU (the EuSOS study), three times as many patients already extubated in there PACU were transferred to the ICU. Although this might reflect the unique risk that caregivers attributed to the complicated perioperative course of these patients, the specific cause needs to be sought in further studies.

Overall, our results reflect the high risk for postoperative morbidity and mortality of this unique patient population that unexpectedly requires postoperative respiratory support. Future investigations should be aimed at identifying these patients before surgery, stratifying their perioperative risk, and eventually making a better-calculated decision on acceptable risk-benefit balance.

Our study suffers several limitations. First, like most observational studies, it is retrospective in nature and is therefore limited in terms of data quality. It should be noted, though, that despite the large cohort size, data were collected by manual chart review of each case by trained anesthesiologists and are therefore presumably as accurate as patients’ medical records. Second, we did not collect data about a control group of patients with normal clinical course and extubation at surgery conclusion. Nevertheless, comparing mortality alone, the reported 30-day mortality among adults undergoing noncardiac surgery is 1–2% [Devereaux et al. 2012, 2017] comparable with a 1% in-hospital mortality rate among all adult surgical patients in our institutions.

## Conclusions

Unplanned postoperative mechanical ventilation is relatively rare but is associated with noteworthy morbidity and mortality, with significantly higher rates in those of need for protracted (vs. short) mechanical ventilation. Remarkably, morbidity, and mortality were notably high even in patients who were extubated shortly after arriving to the PACU. This patient population deserves further investigation to explore contributing factors for non-extubation and for complications and whether we can intervene to improve outcome or alternatively acknowledge and communicate the increased risk.

## Data Availability

The datasets generated and/or analyzed during the current study are not publicly available due to institutional regulations but are available from the corresponding author on reasonable request.

## Abbreviations

PACU: Postanesthesia care unit
ICU: Intensive care unit
TLVMC: Tel-Aviv Sourasky Medical Center in Tel-Aviv
WMC: Edith Wolfson Medical Center in Holon
SD: Standard deviation
IQR: Interquartile range
OR: Operating room
ISOS: International Surgical Outcomes Study
EuSOS: European Surgical Outcomes Study
